# A Miniature Jumping Robot Using Froghopper’s Direction-Changing Concept

**DOI:** 10.3390/biomimetics10050264

**Published:** 2025-04-24

**Authors:** Dong-Jun Lee, Gwang-Pil Jung

**Affiliations:** Department of Mechanical and Automotive Engineering, Seoul National University of Science and Technology, Seoul 01811, Republic of Korea

**Keywords:** bio-inspired robot, jumping robot, direction change, froghopper

## Abstract

To improve the maneuverability and agility of jumping robots, a variety of steerable jumping mechanisms have been actively studied. The steering ability enables a robot to reach a particular target by altering its jumping direction. To make this possible, we propose a miniature steerable jumping robot based on froghopper’s jumping principle: Moment cancellation is achieved via synchronous leg rotation, and a predictable jumping direction is achieved through an almost zero stiffness femoro-tibial joint. To satisfy these working principles, the robot is designed to have a four-bar shaped body structure and wire-driven knee joints. The four-bar body always synchronizes the leg operation by mechanically coupling the two jumping legs, which enables the robot to cancel out the moments and finally reduce the needless body spin. The knee joints are actuated using wires, and the wires are kept loose to maintain joint stiffness almost zero during take-off. Accordingly, the jumping direction is successfully predicted to determine the initial posture of the tibia. As a result, the proposed robot can change the jumping direction from −20 degrees to 20 degrees while reducing needless body spin.

## 1. Introduction

Jumping has been widely utilized for miniature jumping robots to enable them to overcome large obstacles and distances. To achieve strong jumping performance, there have been numerous attempts based on escapement mechanisms using a one-way bearing [1], an inchworm motor [2], a cam or gear [3,4], combustion [5,6], and an active clutch [7,8,9]. These mechanisms have offered miniature jumping robots the benefit of overcoming large obstacles, but most of them have concentrated on jumping locomotion itself.

To improve the maneuverability of miniature jumping robots, several researchers have investigated the effect of adding a steering function [10,11,12,13,14,15]. Miniature jumping robots with steering ability can reach a target by, for example, adjusting their launch direction. Previously, Kovač et al. made a 14 g miniature jumping robot that could steer and upright itself [16]. Steering is achieved by rotating the whole jumping mechanism using a double-guided axis and a DC motor. Stoeter et al. proposed a “Scout robot” that consists of two wheels and a winch [17]. The robot controls its location by rolling on the plane and jumping up to 30 cm into the air. Zhao et al. made a steerable jumping robot that weighs 23.5 g and uses a single DC motor [1]. The robot changes its jumping direction by rotating the gear that contacts the ground first. Weiss et al. presented a 2.5 kg jumping robot employing a piston-driven combustion chamber [18]. The robot’s jumping orientation is controlled by adjusting the location of its center of gravity. Armour et al. presented the “Jollbot”, which consists of metal hoop springs forming a 300 mm diameter sphere [19]. The robot can roll, jump, and change jump direction by adjusting the center of mass of the robot slightly. Burdick et al. utilized an active steering method using a ring gear at the foot of their robot [20]. The whole body, therefore, could rotate around the vertical axis.

These mechanisms have shown successful performance in steering. In some cases, however, their jumping performance has deteriorated a bit due to the increased weight of the additional steering structures. To change the jumping direction without a serious drop in the jumping performance, we previously proposed a novel direction-changing concept adopted from froghoppers’ jumping principles: moment cancellation via synchronized leg operation and a predictable jumping direction based on almost zero stiffness femoro-tibial joints. With these principles, froghoppers hardly show a performance drop in jumping, even though they change their jumping azimuth. To apply froghopper’s jumping principle, the previous concept used a conventional gear to synchronize the leg operation and the freely rotating knee joints to predict the jumping direction. As a result, the mechanism has shown that it has the potential to be used for jumping robots to change and predict the take-off direction while minimizing needless body spin. In terms of real world applications, however, there are still several limitations: (1) the synchronizing mechanism is not reliable since the conventional gears are unstably installed, (2) there is no leg-alternation mechanism to change the jumping direction, (3) the structure is not robust because of the layer-based fabrication method, and (4) a clutching mechanism is required to store and release the energy.

**Figure 1:**
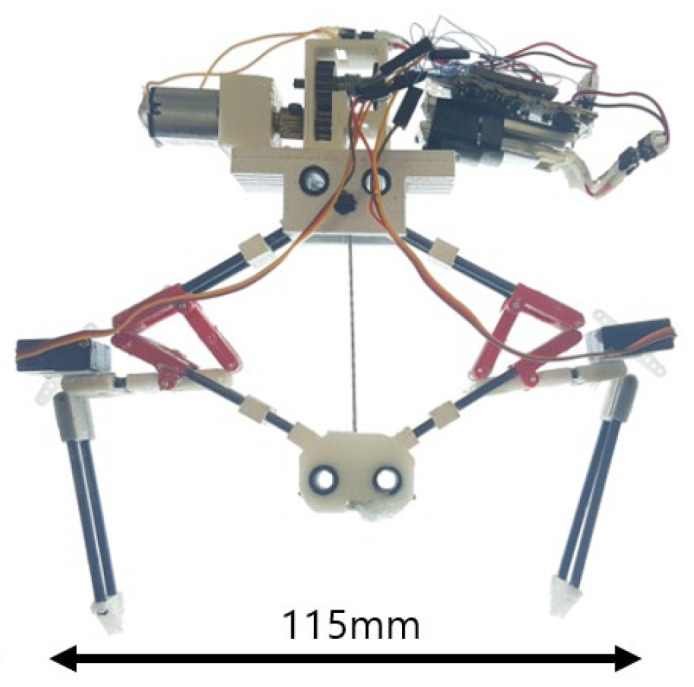
An azimuth-steerable jumping robot, showing the initial positions for forward jump.

In this paper, we suggest solutions to solve the issues and present a milli-scale untethered robot that can steer and jump as shown in Figure 1. The key design components are the four-bar shaped body and the zero-stiffness knee joints, which allow the robot to satisfy froghopper’s working principles and complement the weakness of the previous concept. The four-bar-shaped body replaces the conventional gears and always synchronizes the operation of symmetrically positioned jumping legs through kinematic coupling. This enables the robot to cancel out the moments from the jumping legs and finally reduce needless body rotation. The posture of the tibia is altered using wires connected from the foot to the servomotor on the femur. The wires are kept loose to allow the knee joints to freely rotate during take-off, which enables the successful prediction of the jumping direction. As a result, the robot can change the jumping direction from −20 deg. to 20 degrees without much of a drop in jumping performance.

This paper is organized as follows: The design section describes the concept of the jumping mechanism derived from the froghoppers’ jumping and the working process of the steerable jumping robot. To predict the jumping direction of the robot, free body diagrams of the tibia and the femur are derived. Furthermore, several jumping experiments are done to investigate the jumping performance by varying the jumping azimuth and to prove our jumping concept.

## 2. Design

### 2.1. Froghopper-Inspired Direction Changing Concept

**Figure 2:**
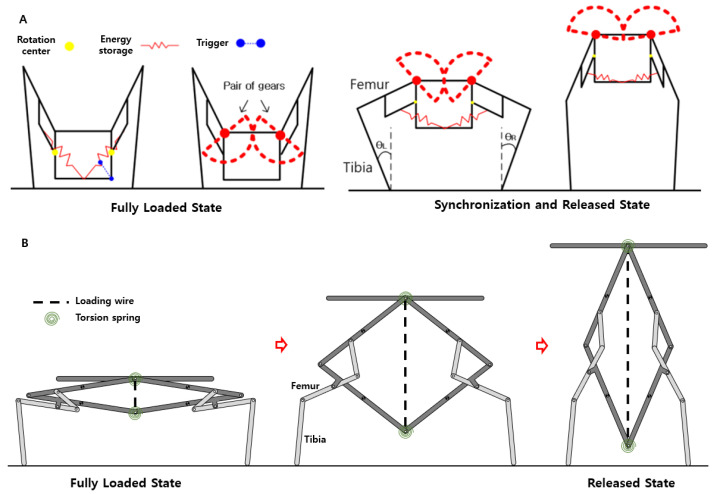
(**A**) Schematic of the previously proposed direction-changing mechanism. (**B**) Schematic of the newly proposed jumping mechanism in the fully levated position (left), in the middle of jumping (mid), and in the fully released position (right).

The froghopper, Philaenus spumarius, is well known for its outstanding jumping performance. The froghopper jumps with a mean velocity of 2.8 ms in 1.2 ms. This corresponds to an average power of 35 W/g [21], which is much larger than that of locust (0.45 W/g). This amazing jumping performance originates from its unique jumping principles: Power-producing hind legs and moment cancellation are achieved due to synchronized leg operation. Thanks to these working principles, the initially stored energy in hind trochanteral depressor muscles effectively transfers to translational kinetic energy, resulting in reduced body spin, as shown in Figure 2A.

Furthermore, the froghopper’s initial posture tells us its real jumping direction [22]. The froghopper has almost freely rotating femoro-tibial joints, and the joints do not transmit any torque to the tibia. Therefore, the direction of the reaction force from the ground corresponds to the posture of the tibia. Based on this information, the jumping direction can be estimated as the average angle of both tibiae.

Inspired by the froghopper’s jumping, we previously proposed a direction-changing concept for miniature jumping robots, as shown in Figure 2B. The proposed mechanism has two power-producing legs with their own energy storage and conventional gears for simultaneous leg operation. As a result, the mechanism could successfully change direction from −40 degrees to 40 degrees, with an improved take-off speed.

### 2.2. Simplified Jumping Mechanism Design

In the previous work, we have shown that the froghopper-inspired direction-changing concept has the potential to be applied to miniature jumping robots. Directly employing the proposed concept in an untethered robotic system for real world applications, however, is not easy because of the following reasons: (1) a reliable synchronizing mechanism is needed for simultaneous leg operation, (2) a leg-alternation mechanism is required to change the jumping direction, (3) the structure must be able to withstand repeated operation, (4) a clutching mechanism is essential to store and release the energy. To solve these issues, we simplify the previous concept using a four-bar shaped body structure, wire-driven knee joints, a simple clutch mechanism, and rolling contact joints for repetitive operation.

Figure 2 shows the detailed design and working process of the previous and current jumping mechanisms. Basically, the current mechanism is designed to always synchronize the operation of the jumping legs so that the moments generated by the legs cancel out each other. To this end, a four-bar-shaped body, a pair of symmetrically positioned jumping legs, and couplers connecting the body with the jumping legs are used, as shown in Figure 2B. The previous mechanism shown in Figure 2A has a pair of gears on both femora. Gears installed on both femora keep the leg motions synchronized at all times. However, depending on the size of the gear teeth, backlash may occur, potentially causing desynchronization of the leg movements. In contrast, the current mechanism uses a four-bar linkage that guarantees one degree of freedom, allowing the leg motion desynchronization to be reduced to nearly zero. When the four-bar folds and unfolds, the jumping legs naturally move along the four-bar due to the couplers. Accordingly, both jumping legs operate simultaneously, resulting in the successful cancellation of moments and the reduced needless body spin in the end. However, in an actual robot, synchronized motion may not be fully achieved due to factors such as material deformation, assembly errors in the rolling-contact joints, and friction between components. To prevent significant timing discrepancies in leg movements, careful attention must be paid to these issues during the robot’s fabrication.

**Figure 3:**
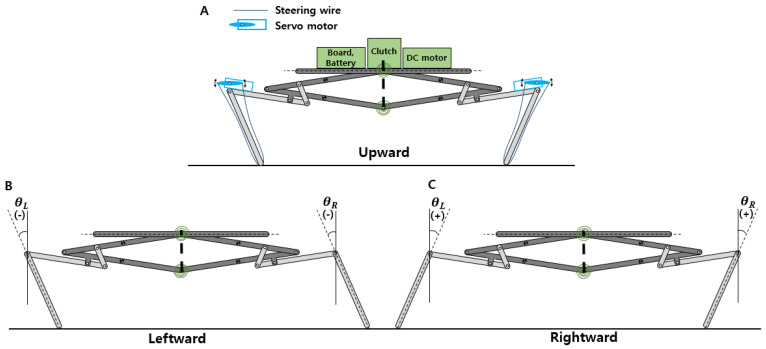
The initial posture for jumping (**A**) upward, (**B**) leftward, and (**C**) rightward.

The initial posture of the jumping legs is controlled using miniature servomotors located at each femur, as shown in Figure 3A. The servomotor has two wires, and the wires are connected to the foot of the jumping leg. One wire pulls the foot in a clockwise direction, and the other pulls in a counterclockwise direction. By using two wires, the angle between the tibia and the ground can be adjusted, as shown in Figure 3B,C.

**Figure 4:**
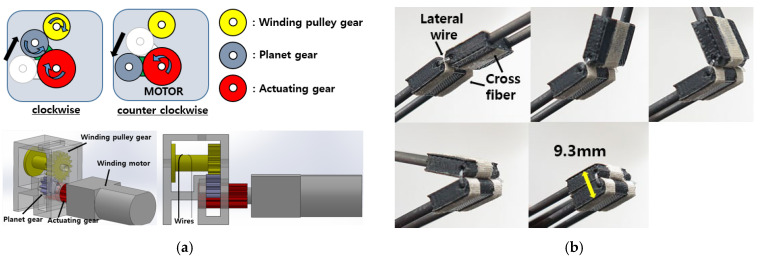
(**a**) Clutch mechanism used to store and release the energy. (**b**) Rolling contact joints to withstand repetitive operation.

The jumping energy is stored in the torsional springs located at the top and the bottom of the four-bar, as shown in Figure 2B. When the four-bar is fully folded by the loading wire, the jumping energy is maximally stored. The energy is stored and released through a clutch mechanism, as shown in Figure 4a. The clutch consists of three gears and a DC motor. When the motor and the actuating gear rotate in a clockwise direction, the planet gear contacts the winding pulley gear and starts to wind the wire to store the energy. As soon as the motor rotates in a counterclockwise direction, the planet gear detaches from the winding pulley gear, and the stored energy is released.

In addition, knee-inspired rolling contact joints are utilized. The rolling contact joints consist of three crossed straps and a lateral wire, as shown in Figure 4b. The lateral wire resists the twisting force, and the crossed straps resist the high compression force. Both the twisting force and the compression force are exerted on the joint when the four-bar begins to fold to store the energy. If the rolling contact joint fails to bear those forces, the four-bar structure folds out-of-plane and, finally, is broken.

Figure 3A shows the description of the jumping robot with all its components. A DC motor, control boards, battery, and clutch mechanism are located at the top of the robot. The main components of the robot are arranged with consideration so that the center of mass is positioned centrally. This helps maintain balance during jumping. The quantity of the components and the corresponding mass ratio of the jumping robot are given in Table 1.

## 3. Jumping Azimuth Analysis

To examine how reaction forces and moments affect the take-off azimuth, the tibia and the femur were analyzed based on free body diagrams and the corresponding equations. To simplify the analysis, the following assumption was made: the femoro-tibial joints have low stiffness, rotate freely, and do not transmit any torque to the tibia.

The posture of the tibia has relevance to the take-off direction since the distal end of the tibia touches ground and the reaction exerts force on it. The direction of this reaction force influences which direction the robot takes off at. Accordingly, the relationship between the reaction force exerted on the tibia and the initial posture of the tibia must be investigated.

**Figure 5:**
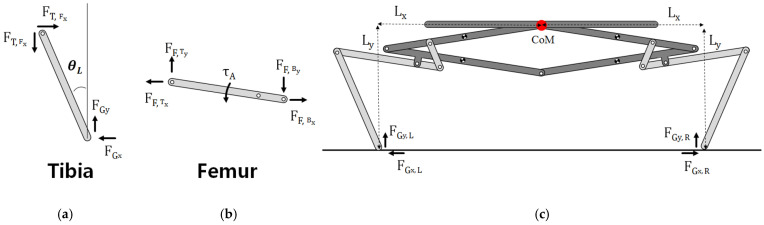
Free body diagrams (FBD) of the body and components. FBD of (**a**) tibia, (**b**) femur, and (**c**) whole mechanism.

To examine the relation between the take-off direction and the initial posture, free body diagrams of the tibia, the femur, and the body are derived in Figure 5. The internal forces cancel out each other based on the principle of action and reaction. Then, the reaction force from the ground only remains as an external force it exerts on the tibia.

Figure 5a indicates the free body diagram of the tibia. In the assumption, the femoro-tibial joint hardly has stiffness and is regarded as a freely rotating joint. Therefore, any torque generated during jumping is not transmitted to the tibia through the femoro-tibial joint, and consequently, the net torque on the tibia becomes almost zero, as follows:

(1) $$L_{tibia}F_{G_{x}}\cos\theta_{L}-L_{tibia}F_{G_{y}}\sin\theta_{L}\approx 0$$

Here, *L_tibia_* denotes the tibial length, *θ_L_* denotes the angle of the left tibia, and *F_Gx_* and *F_Gy_* denote the ground reaction forces in the *x*- and *y*-direction, respectively.

Accordingly, the following relation can be derived:

(2) $$tan\theta_{L}=\frac{F_{G_{x}}}{F_{G_{y}}}$$

Equation (2) tells us that the direction of the reaction force is parallel to the direction of the tibia, like froghoppers [22]. That is, the direction of the reaction force can be estimated by the initial angle of the tibia, which is how the take-off direction can be predicted. Given that an object moves in the direction that is specified by a force vector, accordingly, the take-off direction can be predicted by averaging the direction of force vectors generated by both tibiae:

(3) $$\theta_{Jump\;direction}\approx \frac{\theta_{\;L}+\theta_{R}}{2}$$

The angles *θ_L_* and *θ_R_*, corresponding to the left and right tibiae, respectively, are illustrated in Figure 3B.

In terms of whole robot, reaction forces from the ground only exert external force on the robot. Therefore, the equation of rotating motion for the whole robot is given as follows:

(4) $$I\ddot{\theta }=F_{G_{y},\;\;R}L_{x}+F_{G_{x},\;\;R}L_{y}-F_{G_{y},L}L_{x}-F_{G_{x},\;\;R}L_{y}$$

*L_x_* and *L_y_* are the lengths between the center of mass and the contact point of the tibia and ground, and *F_G,y_*_R_ and *F_Gy,L_* are the ground reaction force of the left and right leg in the *y* direction, respectively.

Since the robot has symmetric configuration, the moments generated by the reaction forces cancel out each other, as shown in Equation (4): the first and the second terms produce the moment in a clockwise direction, while the third and the fourth term cause the moment to occur in a couterclockwise direction. Consequently, the robot can take off with reduced body spin.

## 4. Experimental Results

### 4.1. Method

To evaluate the proposed prediction model for the jumping direction, a series of experiments were conducted with varied initial tibial postures. The steerable jumping robot was designed to adjust its take-off direction by altering the initial angles of the tibiae. Based on the jumping azimuth analysis, it is hypothesized that the take-off direction can be predicted by the average angle between the tibiae and the ground.

In these experiments, the average tibial angle was kept constant, while the individual angles of the left and right tibiae were deliberately varied. The detailed values are provided in Table 2. Although the resulting jumping directions appear similar, each case is defined by a unique combination of tibial angles. Through this design, the robustness of the prediction model was verified, as it was demonstrated that the same average angle leads to similar jumping directions, regardless of asymmetry between the legs.

Jumps in upward, leftward, and rightward directions were tested to cover a full range of motion, and each configuration was repeated three times to confirm repeatability. After validating the hypothesis, the robot was tested under untethered conditions to explore practical applications.

After checking the assumption, the jumping robot was tested for real-world applications. Two servomotors and tendons were installed at each femur to alter the initial angle of both tibiae. A Bluetooth module (Bluno; DF robot, Inc., Shanghai, China) was employed to adjust the initial posture and to trigger the jumping robot wirelessly.

All tests were performed on a 1 mm-thick polymer pad (Dragon Skin; Smooth-On, Inc., Macungie, PA, USA) to ensure sufficient friction and prevent slippage. A high-speed camera (1000 fps) was employed to record the motion, and the jumping kinematics—such as initial tibial posture, take-off direction, translational velocity, and angular velocity—were analyzed using Kinovea software 2023.1.2.

### 4.2. Jumping Direction

To investigate the relationship between the initial average angle and the real jumping direction, the initial angle of the left and the right tibia is set as shown in Table 2. All cases in Table 2 have a difference in the initial angle of each tibia but have an equal average angle. In the case of upward jumping, for example, three cases have different angles for each tibia, but they have almost the same average angle of 0°.

**Figure 6:**
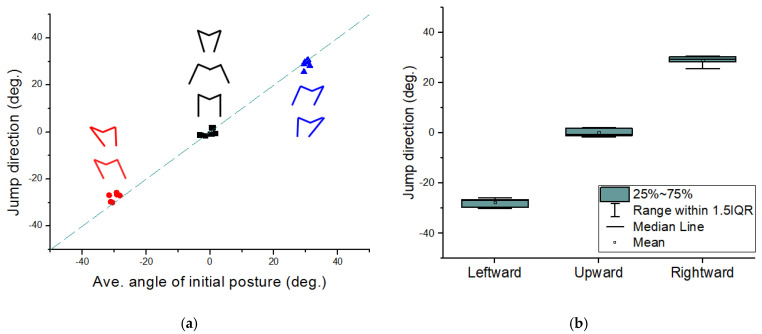
(**a**) Relation between jump direction and average angle of initial posture. The dotted line is a linear function of *y* = *ax*, where *a* = 1. Upward, rightward, and leftward jumps are indicated by black, blue and red lines, respectively. (**b**) A plot of jump direction for each group, showing the median, interquartile range (IQR), and mean.

The experimental results are plotted in Figure 6. Figure 6 shows the jumping direction depending on the average angle of the initial posture. The overall jump direction ranges from −30° to 30°. Basically, the jumping direction shows good agreement with the average angle of the initial posture. For example, the robot jumps rightward when the average of the initial angles has a positive value. To evaluate the consistency of directional jumps, the average initial posture angle and the resulting jump direction were plotted for each target direction: leftward, upward, and rightward. Figure 6b presents the distribution of jump direction data across repeated trials within each group. The results clearly show that each jumping direction forms a compact and well-separated distribution. Notably, the upward and rightward groups display very narrow IQRs, reflecting low trial-to-trial variability in those configurations. The leftward group, while showing slightly more spread, remains tightly clustered with minimal deviation.

**Figure 7:**
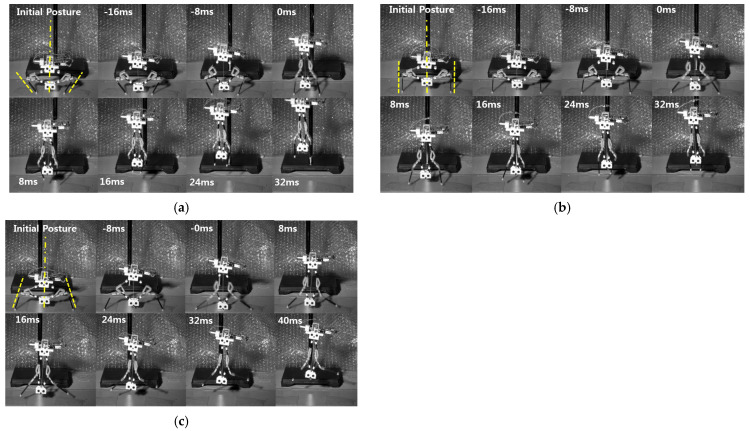
Upward jumping with three different initial postures. The robot has initial angle of (**a**) −37.38° (left) and 34.48° (right), (**b**) 0.7° (left) and 1.06° (right), and (**c**) 17.06° (left) and −16.2° (right). The dashed line indicates the initial direction of the jumping legs, and the dashed dot line indicates the average of the initial direction. 0 ms indicates the moment of take-off.

**Figure 8:**
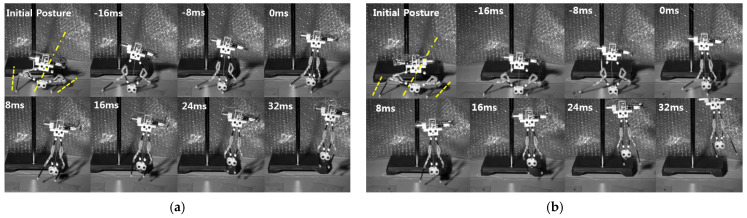
Rightward jumping with two different initial postures. The robot has initial angle of (**a**) 7.84° (left) and 51.0° (right) and (**b**) 22.15° (left) and 39.23° (right). The dashed line indicates the initial direction of the jumping legs, and the dashed dot line indicates the average of the initial direction. 0 ms indicates the moment of take-off.

**Figure 9:**
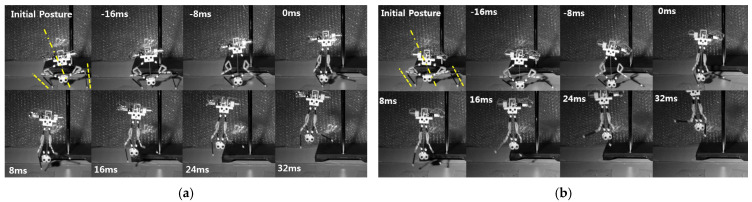
Leftward jumping under two different initial postures. The robot has initial angle of (**a**) −48.25° (left) and −8.26° (right) and (**b**) −28.81° (left) and −32.43° (right). The dashed line indicates the initial direction of the jumpig legs, and the dashed dat line indicates the average of the initial direction. 0 ms indicates the moment of take-off.

Figure 7, Figure 8 and Figure 9 show the moment of jumping with various initial postures. In Figure 7, the initial postures indicate that the robot has an initial average angle of about 0° so that the robot can jump upward. As shown in Figure 7a–c, all cases take off almost upright, even though each tibia has a different initial angle. Figure 8 shows the rightward jumping. Both Figure 8a,b have equal value of the initial average angle of about 30 °, but they have a difference when it comes to each tibia as well. Figure 8a has an initial angle of about 37° and 22° for the left and the right tibia, respectively. In the case of Figure 8b, the left and the right tibia are adjusted to 9° and 50°, respectively. Leftward jumping is similarly performed, as shown in Figure 9. Figure 9a has an initial angle of about −33° and −28 ° for the left and the right tibia, respectively. In Figure 9b, the left and the right tibia are set as about −9° and −48°, respectively. However, it seems that both cases feature jumps in almost equal directions.

### 4.3. Jumping Performance

**Figure 10:**
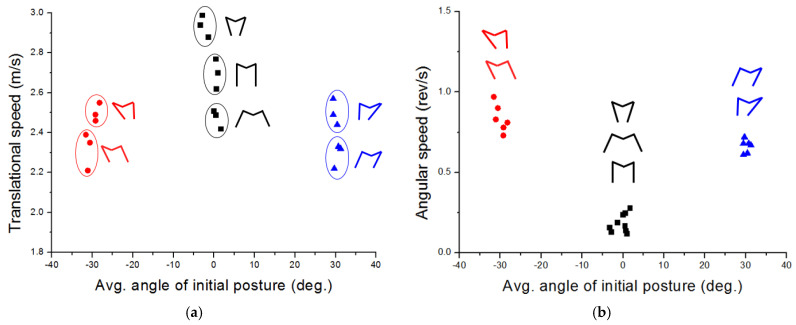
(**a**) Relation between take-off velocity and take-off average angle of initial posture. (**b**) Angular velocity vs. average angle of initial posture. Note that upward, rightward, and leftward jumps are indicated by black, blue and red lines, respectively.

As previously said, the jumping direction is successfully predicted based on the average angle. In this section, we investigate how the take-off direction influences jumping performance by analyzing translational kinetic energy and rotational kinetic energy. Figure 10 shows the relation between translational and rotational speed and the average angle of the initial posture.

In Figure 10a, most of the upward jumping cases show better performance compared to leftward jumping and rightward jumping. One noticeable thing is that the take-off velocity differs depending on the initial posture, even though all upward cases have an equal average angle of initial posture and equal take-off direction. This is due to a difference in the take-off time. In the case of sample No. 1-3 in Table 2, it takes about 31 ms to jump, while case 1-1 takes 25 ms, and case 1-2 takes 21 ms, respectively. Because of the difference in the take-off time, the transferred momentum is maximized in the 1-3 case, and accordingly, the robot shows the highest take-off speed. This phenomenon can also be seen in leftward and rightward jumping. For example, it takes 42 ms to jump for the 3-2 case, while the 3-1 case requires 39 ms for take-off time. As a result, case 3-2 and case 3-1 take off with a speed of about 2.55 m/s and 2.35 m/s, respectively.

Figure 10b shows the angular speed depending on average angle of initial posture. In the case of upward jumping, the robot rarely rotates with an angular speed of 0.11~0.25 rev/s when it jumps. In leftward and rightward jumping, a bit of body spin occurs with an angular speed of 0.8~0.9 rev/s and 0.6~0.7 rev/s for rightward and leftward jumping, respectively.

**Figure 11:**
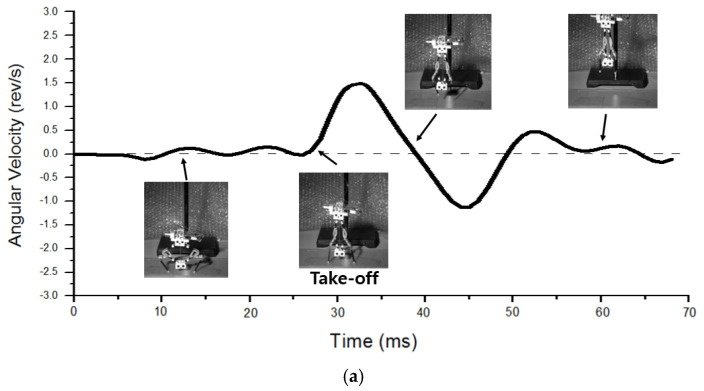

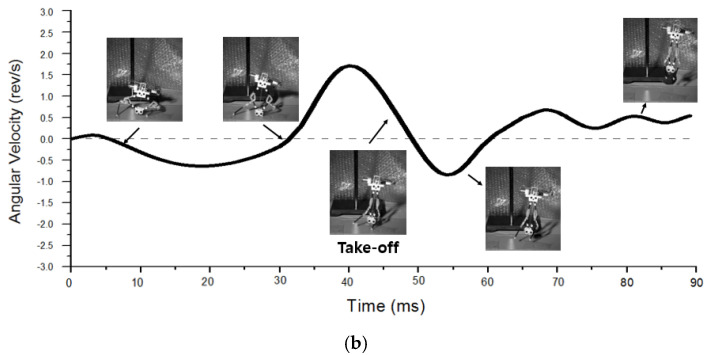
Angular velocity change depending on time for (**a**) upward jumping and (**b**) rightward jumping.

To describe in detail how jumping with reduced body spin occurs, we plotted the angular speed depending on time with the corresponding robot’s posture, as shown in Figure 11. Figure 11a indicates the take-off process of upward jumping case 1-3. When jumping is triggered by the clutch mechanism, the robot hardly shows body rotation before the moment of take-off. Just after take-off, an overshoot of angular speed occurs and instantly converges to near zero. In the case of rightward jumping, the jumping process shows somewhat different behavior. In Figure 11b, the robot’s initial posture is inclined rightward, since the mass center moves to the right. Therefore, when jumping is triggered, the left leg operates first, and the robot starts to rotate in the clockwise direction. Just after the left leg takes off, the robot is affected by a reaction force from the right leg. By doing so, the robot’s body spin is much reduced at the moment of take-off. After take-off, overshoot occurs as well and converges to a certain value of about 0.6~0.7 rev/s.

### 4.4. Application to Jumping Robot

In the previous sections, we confirmed that the jumping direction can be estimated based on the initial posture of the tibiae, and the proposed design allows the robot to take off with reduce body spin. To use the mechanism in real-world applications, an untethered jumping robot was built, as shown in Figure 1. The robot has a servomotor at each femur to control the angle of the tibia. The angle of the tibia is adjusted using wires connected from the servomotor to the foot. After altering the angle, jumping is triggered.

**Figure 12:**
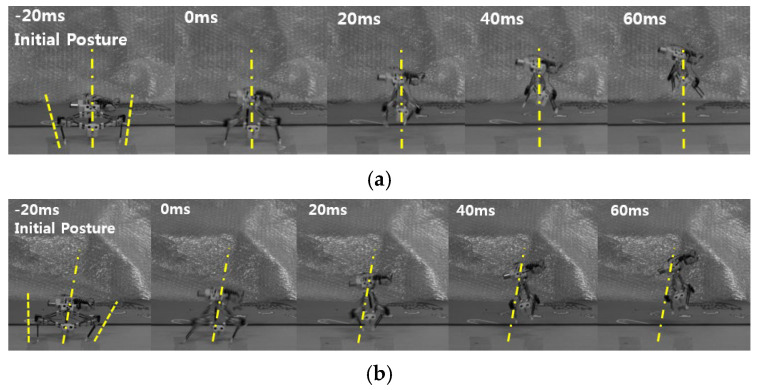

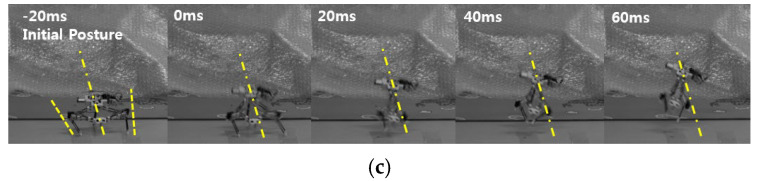
The proposed robot’s jumping with different initial postures. The robot has initial angle of (**a**) −15° (left) and 12° (right), (**b**) −2° (left) and 30° (right), and (**c**) −32° (left) and −5° (right). The dashed line indicates the initial direction of the jumping legs, and the dashed line means the average of the initial direction. 0 ms indicates the moment of take-off.

Figure 12 shows the untethered jumping of the steerable jumping robot. In Figure 12a, the robot has an initial angle of −15° and 12° for the left tibia and the right tibia, respectively. The average value is −1.5°, and the real jumping direction is about −3°. Figure 12b shows rightward jumping. The robot has an initial angle of −2° and 30° for the left and the right tibia, respectively. The robot takes off with a jumping direction of 11°, which is similar to the average angle of 14°. In the case of leftward jumping, the robot takes off in the direction of −17°, as shown in Figure 12c. The robot has initial angles of −32° and −5° for the left and right tibia, respectively. Then, the average value is 18.5° and shows good agreement with the predicted value.

In terms of body spin, however, the robot tends to rotate a bit compared to the previous jumping experiments. This is due to the lack of friction between the foot and the ground. In the case of the untethered robot, the body is inclined, as shown in Figure 1, since the mass center is located on the top of the robot. Therefore, the probability of slipping horizontally increases, and securing enough friction is important for the robot. To this end, a saw-like foot is used, but a slight slip still occurs, as shown in Figure 12.

## 5. Conclusions

In this paper, we propose a steerable jumping robot based on froghopper’s jumping principles: moment cancellation using synchronized leg operation and a predictable jumping direction through an almost zero-stiffness femoro-tibial joint. These principles were applied via a four-bar-shaped body and wire-driven knee joints. This design enables the jumping legs to operate in sync, and accordingly, the moments caused by the legs cancel each other out. As a result, the robot can take off in various directions while reducing unnecessary body spin. Also, by keeping the knee joints loose through a wire-driven mechanism, the jumping direction can be successfully predicted based on the initial posture of the tibiae. To prove the robot’s solid design, several jumping experiments were performed by varying the initial posture of the robot. We confirmed that the robot tended to jump in the direction predicted by the average angle of the left and the right tibiae. In terms of jumping performance, the robot could take off with reduced body spin, even when it jumped leftward and rightward. In addition, an untethered robot was created to show its potential to be used in real-world applications.

## Figures and Tables

**Table 1:** Mass budget.

Components	Quantity (ea.)	Mass (g)	Ratio to Overall Mass (%)
Clutch	1	4.4	8.4
Jumping motor	1	10.1	19.3
Steering motor	2	4.0	7.6
Battery	2	4.8	9.2
Control board	1	4.2	8.0
Body structure	1	16.8	32.1
Torsion spring	4	2.4	4.6
Femur	2	2.6	5.0
Tibia	2	1.8	3.4
Coupler	2	1.2	2.3
Total	-	52.3	100.0

**Table 2:** Initial posture of jumping legs.

Upward Jump									
**Initial Posture**	*** 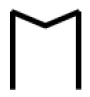 ***			*** 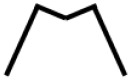 ***			*** 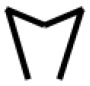 ***		
**Sample No.**	**1-1**			**1-2**			**1-3**		
**Experiment No.**	**1**	**2**	**3**	**1**	**2**	**3**	**1**	**2**	**3**
Initial angle of right leg (deg)	1.06	2.01	2.16	−16.21	−15.20	−18.31	32.59	31.51	34.48
Initial angle of left leg (deg)	0.70	−1.20	−1.05	17.06	15.01	15.02	−39.38	−37.31	−37.38
Avg. angle (deg)	0.88	0.405	0.55	0.43	−0.095	−1.64	−3.395	−2.90	−1.45
Rightward Jump									
Initial Posture	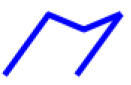			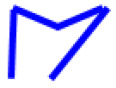					
Sample No.	3-1	3-2							
Experiment No.	1	2	3	1	2	3			
Initial angle of right leg (deg)	39.23	36.25	38.24	7.84	9.80	10.50			
Initial angle of left leg (deg)	22.15	24.54	21.11	51.02	49.01	52.01			
Avg. angle (deg)	30.69	30.39	29.68	29.43	29.40	31.25			
Leftward Jump									
Initial Posture	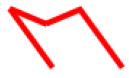			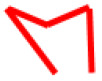					
Sample No.	2-1	2-2							
Experiment No.	1	2	3	1	2	3			
Initial angle of right leg (deg)	−32.43	−33.41	−33.34	−8.26	−10.21	−9.15			
Initial angle of left leg (deg)	−28.81	−29.80	−28.81	−48.25	−48.31	−49.31			
Avg. angle (deg)	−30.62	−31.61	−31.12	−28.255	−29.26	−29.23			

## Data Availability

Data are contained within the article as figures and tables.
